# Genetic Loss of Murine Pyrin, the Familial Mediterranean Fever Protein, Increases Interleukin-1β Levels

**DOI:** 10.1371/journal.pone.0051105

**Published:** 2012-11-30

**Authors:** Pamela R. Hesker, MyTrang Nguyen, Martina Kovarova, Jenny P.-Y. Ting, Beverly H. Koller

**Affiliations:** 1 Curriculum of Genetics and Molecular Biology, University of North Carolina, Chapel Hill, North Carolina, United States of America; 2 Department of Genetics, University of North Carolina, Chapel Hill, North Carolina, United States of America; 3 Department of Medicine, University of North Carolina, Chapel Hill, North Carolina, United States of America; 4 Department of Microbiology and Immunology, University of North Carolina, Chapel Hill, North Carolina, United States of America; 5 Lineberger Comprehensive Cancer Center, University of North Carolina, Chapel Hill, North Carolina, United States of America; University of California Merced, United States of America

## Abstract

Familial Mediterranean Fever (FMF) is an inherited autoinflammatory disorder characterized by unprovoked episodes of fever and inflammation. The associated gene, *MEFV* (Mediterranean Fever), is expressed primarily by cells of myeloid lineage and encodes the protein pyrin/TRIM20/Marenostrin. The mechanism by which mutations in pyrin alter protein function to cause episodic inflammation is controversial. To address this question, we have generated a mouse line lacking the *Mefv* gene by removing a 21 kb fragment containing the entire *Mefv* locus. While the development of immune cell populations appears normal in these animals, we show enhanced interleukin (IL) 1β release by *Mefv* ^−/−^ macrophages in response to a spectrum of inflammatory stimuli, including stimuli dependent on IL-1β processing by the NLRP1b, NLRP3 and NLRC4 inflammasomes. Caspase-1 activity, however, did not change under identical conditions. These results are consistent with a model in which pyrin acts to limit the release of IL-1β generated by activation and assembly of inflammasomes in response to subclinical immune challenges.

## Introduction

Mutations in *MEFV* predispose humans to Familial Mediterranean Fever (FMF), a disease characterized by spontaneous activation of the innate immune system in the absence of a detectable pathogenic stimulus. Fever and acute abdominal pain are the most common symptoms, but disease manifestations also include arthritis, pleuritis, localized erythema, and amyloidosis of the kidneys. The inflammation observed in FMF patients is characterized by neutrophil influx to peripheral tissues and increased serum levels of acute-phase reactant proteins and cytokines [1]. Pharmacological inhibitors of IL-1β/IL-1 receptor signaling are efficacious in the treatment of some patients, supporting the hypothesis that pathophysiology of FMF is mediated in part by this cytokine [2].

*MEFV* encodes a 781 a.a. cytoplasmic protein comprised of 5 domains: pyrin, b-Zip, B-Box, coiled-coiled, and PRY/SPRY (B30.2), although this later domain is absent in the mouse protein. The pyrin domain is of particular interest since this domain is found in several proteins critical in IL-1β production [3]. Similar to most cytokines, *Il1β* transcript levels are enhanced by cellular stimulation with cytokines or pathogen-associated molecular patterns (PAMPs) [4]. However, the *Il1β* gene does not encode a leader peptide to facilitate transport and secretion of the protein [5]. Instead, it is synthesized as an inactive pro protein which is cleaved by the cysteine-aspartic acid protease caspase-1 (CASP-1) to form mature IL-1β. Catalytic activity of CASP-1 also requires removal of a regulatory domain, although in this case, this is achieved through autocatalytic cleavage. Activation of CASP-1 is observed after exposure of cells to both host-derived signals of cellular stress or damage, called danger-associated molecular patterns (DAMPs), and to PAMPS. These signals trigger the assembly of inflammasomes, protein scaffolds nucleated by a nucleotide-binding domain, leucine-rich repeat family (NLR) protein. A number of NLRs are capable of assembling an inflammasome in response to unique but overlapping DAMPS and PAMPS. To date, these include NLRP3, NLRP1b, NLRC4, NLRP2 and NLRP6 [6]–[11]. However, assembly of a CASP-1 activating inflammasome complex is not restricted to the NLR family, as more recently, the protein absent in melanoma 2 (AIM2) has been shown to contribute to CASP-1 activation and IL-1β release in response to dsDNA [12].

In addition to the NLR/AIM2 protein, the inflammasome often contains the protein adaptor ASC which facilitates the recruitment of pro-caspase-1 to the complex [3]. Interaction between the pyrin and card domains of various proteins is critical for inflammasome assembly, both the self-association of the NLR proteins and the recruitment of ASC and CASP-1 to the complex. It is, therefore, not surprising that it has been suggested that proteins which contain a PYD or CARD domain, yet lack other critical functional domains, act as negative regulators of inflammasome assembly [13]. Furthermore, a number of studies indicate that pyrin might function in a similar manner, with disease-associated mutations resulting in reduced ability to limit inflammasome assembly [14]-[17]. Such a model is consistent with the recessive pattern of inheritance of the disease [18], [19] and with previous findings that pyrin can bind ASC, CASP-1, NLRPs 1-3, and IL-1β [15], [17], [20], [21].

Not all reports, however, support this model. Molecular genetic reports of both the absence of individuals carrying null *MEFV* alleles and the observation of disease in some individuals believed to carry a wild-type allele, have led to the suggestion that FMF is an autosomal dominant disease with variable penetrance [22], [23]. It has been posited that pyrin itself can assemble a CASP-1 activating inflammasome, with the assembly of the mutant pyrin inflammasome triggered by lower, subclinical, levels of PAMPs/DAMPs [20], [24]–[27]. Transfection and knockdown studies provide limited clarification of the function of pyrin. In some studies, pyrin increased IL-1β release, while in other studies using similar strategies, cytokine production was decreased [14]–[17], [20], [25]–[27]. Furthermore, previous reports that *MEFV* expression is either increased, decreased, or unchanged in FMF patients compared to healthy controls complicates interpretations of the function of pyrin in both homeostatic and inflammatory contexts [28].

To further examine the function of pyrin, we describe here the generation of a mouse line carrying a null *Mefv* allele. This mouse lacks the entire 21 kb locus. Using primary cells from this mouse line, we show that pyrin negatively regulates IL-1β production in response to inflammasome-dependent stimuli.

## Materials and Methods

### Mice

All experiments were conducted in accordance with the National Institutes of Health Guide for the Care and Use of Laboratory Animals. The protocol was approved by the Institutional Animal Care and Use Committee guidelines of the University of North Carolina at Chapel Hill (Permit Number: 10-181).

A targeting vector designed to remove the entire coding region of *Mefv* was assembled from bacterial artificial chromosome bMQ133e1 (Geneservice, United Kingdom) that contains the *Mefv* genomic region of the mouse strain 129S7/SvEv. Bam HI and Xba I restriction digest sites were added at the 3′ end of the Neo^r^ cassette. The targeting vector was introduced into 129S6 derived embryonic stem cells. Chimeric mice were mated with 129S6 mice. Bam-HI digested DNA was assessed by Southern blot analysis using a 3′ probe created by PCR (primers: 5′- GTGGTGGGTCTTCCTGTG -3′ and 5′- GGTCTCTGATGGGGTTGAAA -3′) and a deleted region (DR) probe (primers: 5′-GCAGGGAGGCTTCAAGGACTTTAC -3′ and 5′- TCTCCTCCCCAATCAACGATGTTT -3′). For northern blot, RNA was isolated from recruited peritoneal neutrophils using RNA Bee (Tel-test) and hybridized with [α-^32^P]dCTP-labeled full-length *Mefv* and *β-actin* cDNA. Mice were genotyped by PCR (primers: endogenous: 5′- CTTTGGAGATTGCTGGCTGT -3′, targeted: 5′- AAATGCCTGCTCTTTACTGAAGG -3′, common: 5′- TCCAGGAAATGGAGAGATGG -3′).

*Casp-1* ^−/−^ mice were generated as previously described [29].

### Real-time PCR Analysis

RNA was harvested from bone marrow-derived macrophages (BMMΦ), resident peritoneal macrophages (rpMΦ), and elicited peritoneal macrophages (pMΦ). Total RNA was converted to cDNA using High Capacity cDNA Reverse Transcription Kit. TaqMan universal PCR master mix and recommended probes were used to detect fluorescence by an ABI Prism 7900HT detection system (Applied Biosystems). The probe for *Mefv* (Mm00490258_m1) detects all known splice variants. Gene expression was calculated as the relative expression of *Mefv*, *Il1β*, or *Il6* normalized by *Gapdh* or *18s*.

### *In vitro* Macrophage Activation Studies

Peritoneal cells were collected from mice by lavage and cultured in RPMI media (Gibco) containing 10% FBS (Cellgro), 10 mM HEPES (Gibco), L-glutamine, pen/strep, and 2β-mercaptoethanol at 10^5^ cells/well in a 96-well plate. Cells were activated as follow:

*ATP, flagellin, muramyl dipeptide (MDP), TiO_2_:* cells were primed with 1 µg/ml LPS from *Escherichia coli* (Sigma) for 6 h follow by stimulation with 1mM ATP for 30 min, 20 ng/ml flagellin for 2 h, 250 ng/ml each of lethal factor and protective antigen from *Bacillus anthracis* (LT) for 3 h, or 10 µg/ml MDP and 5 µg/ml titanium dioxide TiO_2_ for 24 h.

*Aluminum hydroxide (Alum), silica, calcium pyrophosphate dihydrate (CPPD):* cells were incubated with 250 µg/ml Alum, 250 µg/ml silica, or 100 µg/ml CPPD, together with 1 µg/ml LPS for 6 h.

Cell culture supernatants were collected and stored at −80°C. After collection of media, cells were lysed and total protein was measured.

### Protein Analyses

IL-1β (eBioscience), IL-6 (eBioscience), and IL-18 (MBL International) ELISA, lactate dehydrogenase, a marker for cell death (LDH, Clontech) and total protein (BCA protein assay, Thermo Scientific Pierce) protocols were followed as recommended by the manufacturer. For western blot analysis, protein was quantified by BCA assay (Thermo Scientific Pierce). Protein was separated by SDS/PAGE, transferred onto a PVDF membrane, and incubated with IL-1β (R&D Systems) or CASP-1 (Upstate) antibodies.

### *In vivo* LPS

Mice were treated with 2.5 mg/kg or 0.25 mg/kg of LPS (serotypes 127:B5 and 055:B5, respectively) by *i.p.* injection. Body temperature was measured with a rectal thermometer probe. Mice were sacrificed at 20 h (2.5 mg/kg of LPS) or 24 h (0.25 mg/kg of LPS). Weight loss was calculated using body weights measured at 0 h and the time of harvest. The peritoneal cavity was lavaged with 4 ml of PBS. Peritoneal cell concentrations were assessed using a hemocytometer. Peritoneal lavage fluid was used to determine IL-1β and TNFα concentrations by ELISA (BD Biosciences) and myeloperoxidase (MPO) by colorimetric assay.

### FACS Analysis of Caspase-1 activity *in vivo*

*Mefv^+/+^* and *Mefv^−/−^* mice were dosed intratracheally with 10 ug *Bacillus anthracis* lethal toxin (LT) or PBS vehicle. Bronchoalveolar lavage (BAL) fluid was collected 2 h after intratracheal instillation. The vast majority of cells in the BAL were considered macrophages based upon morphological criteria. Cells of the BAL were stained with a fluorescently labeled CASP-1 inhibitor, FAM-YVAD-FMK, according to manufacturer’s instructions (Cell Technology). FACS analysis was performed using a Beckman Coulter CyAn ADP Analyzer. Data were analyzed using FloJo (TreeStar) software to determine the geometric mean of fluorescence intensities.

## Results

### Generation of *Mefv* Null Mice and Characterization of Immune Cell Populations

**Figure 1:**
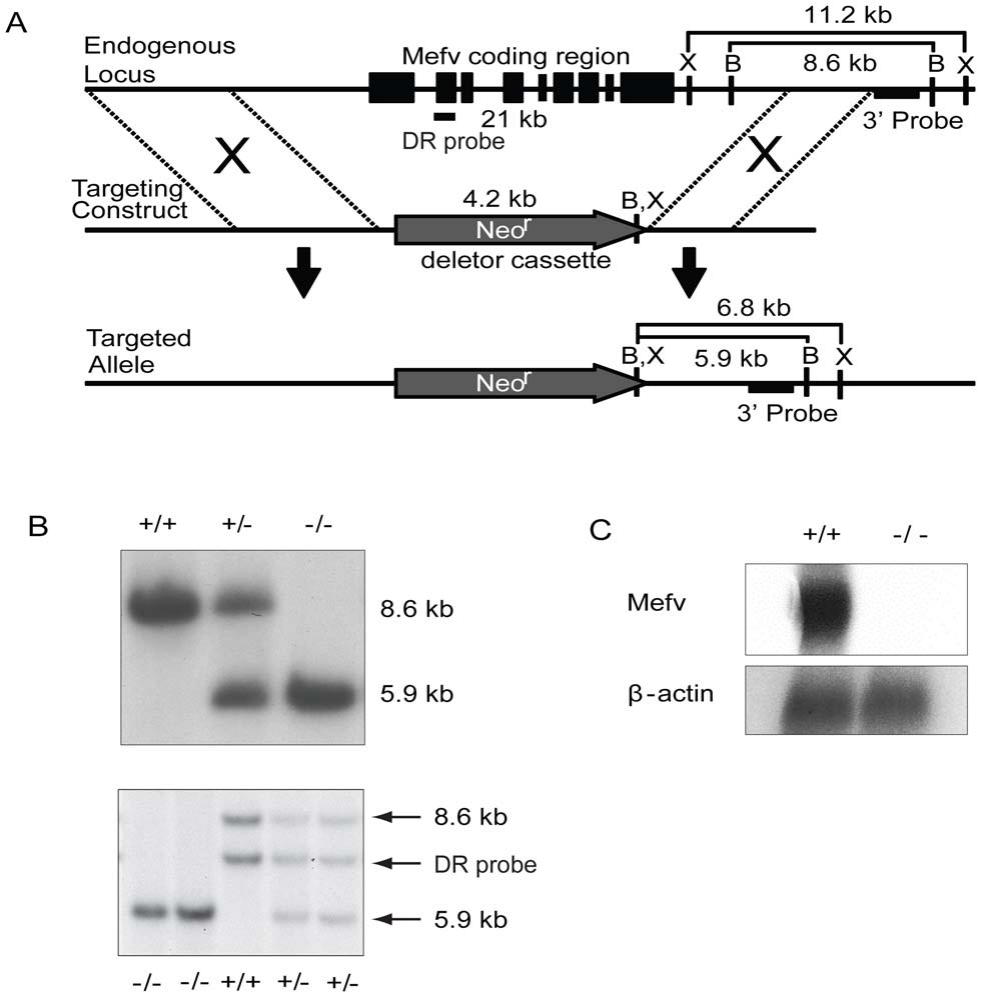
Generation of *Mefv* ^−/−^ mice. *A*, Schematic showing the endogenous *Mefv* locus, gene-targeting construct, and targeted locus after homologous recombination with the targeting construct. Upon homologous recombination, the entire coding region and 5 kb on either side of the *Mefv* gene was replaced with a selectable neomycin resistance (neo^r^) gene. Exons are depicted as filled boxes. The genomic fragment used in Southern blot analysis is indicated as the 3′ probe. *B*, Southern blot was used to screen offspring generated by the intercross of *Mefv* ^+/−^ mice. The 3′ probe detects a BamHI restriction fragment that is diagnostic of proper integration of the targeting construct at the endogenous *Mefv* locus (upper panel). The deleted region (DR) probe corresponds to DNA in exon 2 and identifies a region deleted during homologous recombination (lower panel) *C*, Northern blot analysis of RNA from *Mefv* ^+/+^ and *Mefv* ^−/−^ mice. A full-length *Mefv* cDNA probe was used to detect endogenous expression of *Mefv* in elicited peritoneal neutrophils. Several bands were detected in the WT sample, which is consistent with the presence of multiple splice variants of *Mefv*. The two most intense bands are shown. All bands are absent in *Mefv* ^−/−^ mice. A probe specific to *β-actin* confirms equal loading of the RNA samples.

To generate a mouse line carrying a null *Mefv* allele, we designed a targeting construct capable of deleting the entire *Mefv* locus. Homologous recombination between this vector and the endogenous gene resulted in deletion of all *Mefv* exons, the 5 kb promoter region, and the intergenic region immediately 3′ of the 3′UTR (Figure 1a). Chimeras generated from targeted 129S6 ES cell lines were crossed with 129S6 females to maintain the null allele on this inbred genetic background. Inheritance of the targeted locus was detected by Southern blot (Figure 1b) and followed a Mendelian pattern. DNA from animals homozygous for the targeted locus (*Mefv^−/−^*) was subjected to additional Southern blot analysis to verify absence of the *Mefv* gene. As expected, DNA from these pups failed to hybridize with probes corresponding to various regions of the *Mefv* gene (Figure 1b and data not shown). Moreover, *Mefv* transcripts could not be detected, either by real-time PCR or northern blot analysis, in RNA prepared from elicited peritoneal neutrophils of *Mefv* ^−/−^ animals (Figure 1c and data not shown).

**Figure 2:**
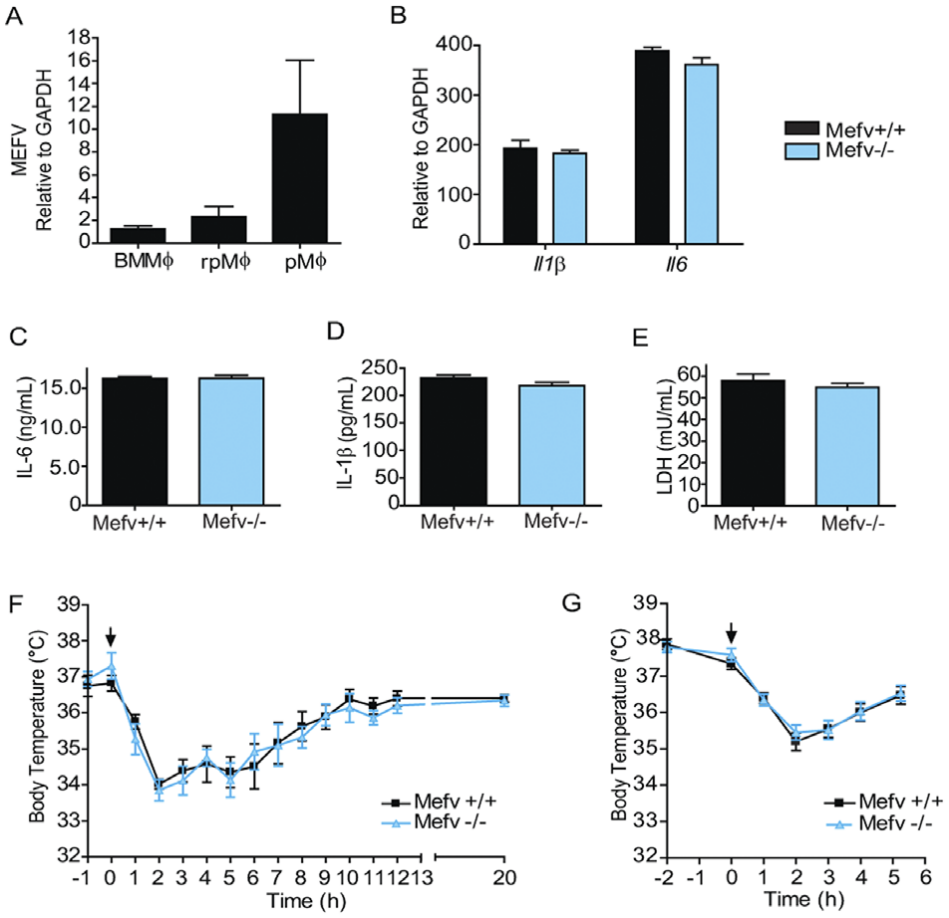
Loss of Mefv does not affect the response to LPS. *A*, Relative expression of *Mefv* in bone marrow-derived macrophages (BMMΦ), resting peritoneal macrophages (rpMΦ), and elicited peritoneal macrophages (pMΦ) from WT mice was detected by real-time PCR. n ≥3 mice per cell type. Data are expressed as relative expression normalized to *Gapdh* and BMMΦ. *B-D*, rpMΦ from *Mefv* ^+/+^ and *Mefv* ^−/−^ littermate mice were untreated or treated with 1 µg/mL LPS for 24 h. *B*, Il1β and Il6 expression at the transcript level is expressed as fold induced by LPS treatment. n = 3 mice per genotype. *C-E*, Concentration of IL-6 and IL-1β in supernatants from LPS-treated rpMΦ cultures as determined by ELISA. n = 6 mice per genotype. *E*, The concentration of lactate dehydrogenase (LDH) released into cell supernatants was used as an indicator of cell death. WT and *Mefv* ^−/−^ mice were treated with 2.5 mg/kg (*F*, n = 8 WT and 5 *Mefv* ^−/−^ mice) or 0.25 mg/kg (*G,* n = 18 and 19) crude LPS by i.p. injection at time 0 h, as indicated by arrows. Rectal temperature at the indicated times is shown in *F* and *G*.

Loss of *Mefv* expression did not impact the survival, growth, or reproductive performance of the mice, nor could *Mefv* ^−/−^ mice be distinguished from littermates based on morphological or behavioral criteria. No change in size and cellularity of the thymus, spleen, or lymph nodes was apparent. Furthermore, analysis of the cellular composition of these organs failed to identify a role for *Mefv* in the development of lymphocytes or cells of myeloid lineage (Table 1 and data not shown), despite the high level of expression of *Mefv* in this later population. *Mefv* expression was easily detected in bone marrow-derived macrophages. However, *Mefv* expression was higher in resident peritoneal macrophages (rpMΦ) and the expression is markedly induced in peritoneal macrophages collected from the peritoneal cavity 72 hours after the induction of peritonitis with the sterile immunostimulant, thioglycolate (Figure 2a). Loss of *Mefv* did not alter the numbers of resident macrophages or the recruitment of macrophages to the peritoneal cavity in response to these inflammatory stimuli (data not shown).

**Table 1:** Analysis of blood collected from *Mefv* ^+/+^ and *Mefv* ^−/−^ mice.

Genotype	*Mefv* ^+/+^	*Mefv* ^−/−^
**WBC** (10^3^/µl)	1.71±0.28	2.12±0.35
**RBC (10^6^/µl)**	8.29±0.16	8.34±0.24
**PLT (10^3^/µl)**	238.1±29.55	285.5±31.61
**LYMPH (%)**	73.47±3.29	67.25±6.08
**GRAN (%)**	17.08±2.13	23.72±0.65
**MONO (%)**	10.50±0.65	10.03±0.65
**HCT (%)**	45.29±0.98	45.73±1.09
**HGB (g/100 ml)**	13.79±0.25	13.90±0.30

Blood was collected by cardiac puncture. Whole blood containing 5 mM EDTA was used for analysis on the Heska Hematology Analyzer by the Animal Clinical Chemistry and Gene Expression Laboratory at UNC-Chapel Hill. n = 13 and 13 mice. **WBC**- white blood cells, **RBC**- red blood cells, **PLT**- platelets, **LYMPH** – lymphocytes, **GRAN** – granulocytes, **MONO** – monocytes, **HCT** - hematocrit, **HGB** hemoglobin. Differences between Mefv^+/+^ and Mefv^−/−^ were not statistically significant.

### Pyrin-deficient Mice Displayed Normal Responses to LPS

The N-terminal cleaved fragment of pyrin has been reported to interact with IκB-α and the p65 subunit of NF-κB, increasing the activity of this transcription factor [30]. If this model is correct, it is reasonable to expect that the induction of NF-κB transcripts after treatment of cells with inflammatory mediators such as LPS would be attenuated in *Mefv* ^−/−^ mice. To test this hypothesis, we compared the mRNA levels of two NF-κB sensitive transcripts, *Il1β* and *Il6*, in macrophages before and after exposure to LPS. Basal levels of mRNA for both of these cytokines were similar in the *Mefv* ^−/−^ and control macrophages. As expected, treatment of the cells with LPS for 24 hours resulted in a robust increase in expression of these genes. However, the magnitude of the increase in expression of *Il1β* and *Il6* was not altered in cells lacking pyrin (Figure 2b).

To address this point further, we examined the level of IL-6 and IL-1β in the supernatant of cultures of rpMΦ before and after LPS treatment. IL-6, similar to most cytokines, is released through a classical secretion pathway [4]. Consistent with the increase in mRNA levels, a dramatic increase in the level of IL-6 was observed in the supernatants from the LPS-treated cells. No difference, however, was observed in IL-6 levels in samples from wild-type (WT) and pyrin-deficient cell cultures (Figure 2*C*). Similar to IL-6, LPS results in an increase in expression of *Il1β* mRNA, but efficient release of this cytokine requires processing of the immature protein by CASP-1 [7]. Thus, not unexpectedly, the level of IL-1β in the supernatant collected from macrophage cultures is low, and while these levels increased significantly after LPS treatment, the magnitude of this increase was small. Loss of pyrin did not augment or attenuate the release of IL-1β by rpMΦ either left untreated or following treatment with LPS (Figure 2*D*). The presence of cytoplasmic enzyme lactate dehydrogenase (LDH) in the cell supernatant can be used to quantitate cell death [31], [32]. Using this criteria, survival of the macrophages, either under control conditions or in the presence of LPS was not altered by the lack of pyrin: levels of the cytoplasmic enzyme LDH in the supernatant did not differ between samples collected from *Mefv* ^−/−^ and WT cultures (Figure 2*E*).

To further examine the impact of the loss of pyrin on the response to endotoxin, we determined the impact of systemic exposure of *Mefv*-deficient mice to LPS. At high doses, exposure to endotoxin results in hypothermia, which is at least in part dependent on elevated levels of NF-κB-sensitive cytokines such as TNFα [14]. *I.p.* injection of LPS resulted in a rapid drop in temperature, with core body temperature reaching a minimum at 2 h after treatment. Both the magnitude of the drop in temperature, as well as the kinetics of the temperature change and subsequent recovery of the animals was similar in the *Mefv* ^−/−^ and control cohorts. No difference was observed in the cryogenic response of the *Mefv* ^−/−^ and wild-type control animals to *i.p.* LPS at both doses of endotoxin examined (Figure 2*F and G*). In addition to hypothermia, LPS treatment results in a measurable drop in body weight accompanied by local neutrophilic inflammation. Neither a change in body weight or the number and composition of cells recruited to the peritoneal cavity distinguished the *Mefv* ^−/−^ mice from littermate control animals (Table 2).

**Table 2:** Inflammatory response in *Mefv* ^+/+^ and *Mefv* ^−/−^ mice after LPS treatment.

Genotype	LPS dose (mg/kg)	Weight loss (%)	Peritoneal cells (10^6^)	MPO (ng/ml)
***Mefv*** **^+/+^**	2.5	5.74±0.5	38.18±3.4	133.0±6.07
***Mefv*** **^−/−^**	2.5	5.20±0.61	36.45±3.2	138.3±10.54
***Mefv*** **^+/+^**	0.25	5.54±0.65	3.27±0.35	n.a
***Mefv*** **^−/−^**	0.25	5.02±0.44	4.12±0.64	n.a

The body weight lost was measured between 0 h and 20 h post-LPS treatment, immune cells present in the peritoneal lavage fluid, and MPO activity as a measure of neutrophil presence in lavage samples were evaluated 20 h post-LPS treatment. For body temperature changes, see Figure 2F, G. **n.a**. not analyzed. n = 18. Differences between Mefv^+/+^ and Mefv^−/−^ mice within each dose of LPS treatment were not statistically significant.

### NLRP3 Inflammasome-dependent IL-1β Production is Increased in *Mefv*-deficient Macrophages

**Figure 3:**
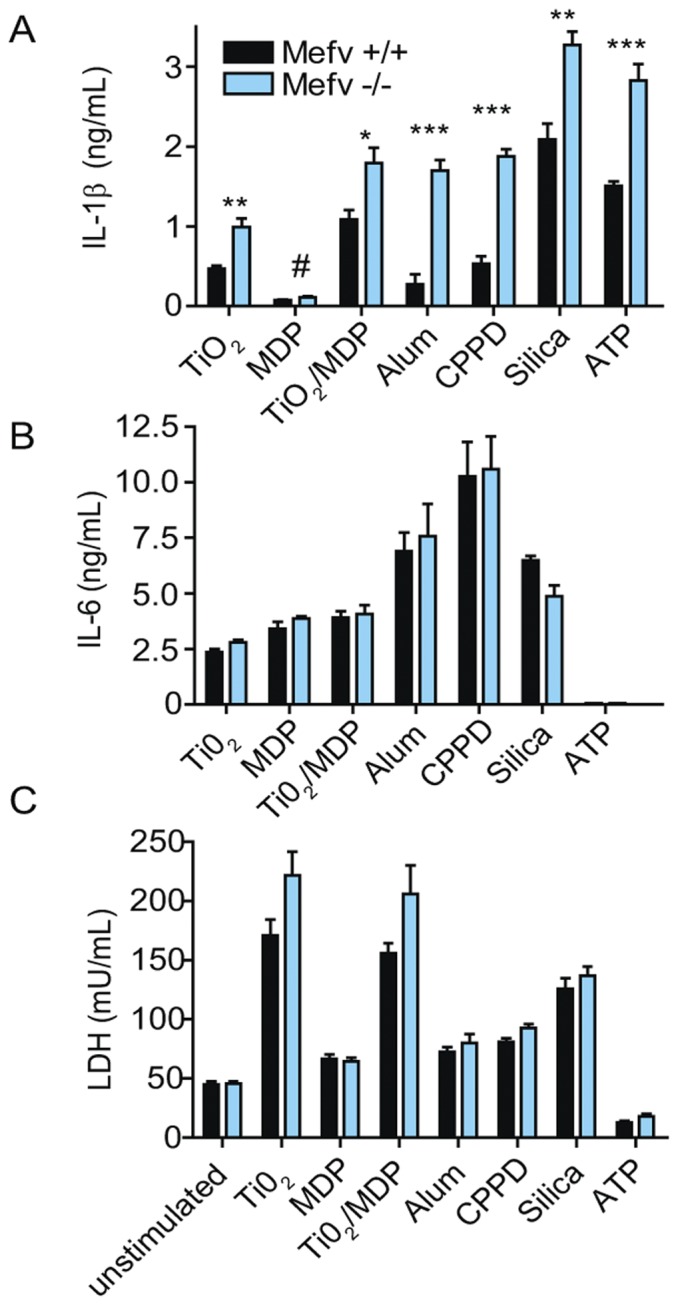
A loss of pyrin causes increased IL-1β protein levels in response to NLRP3 inflammasome stimuli. rpMΦs from *Mefv* ^+/+^ and *Mefv* ^−/−^ littermate mice were exposed to LPS and the indicated stimuli as described in the method section. The concentration of IL-1β, *A*, and IL-6, *B*, cytokines in cell culture supernatants was determined by ELISA. *C*, LDH protein levels detected in cell supernatants. The total protein of cell lysates was measured to verify equivalent plating of cells. TiO2, titanium dioxide; MDP, muramyl dipeptide; Alum, aluminum hydroxide; CPPD, calcium pyrophosphate dihydrate; ATP, adenosine 5′ triphosphate. n = 6 mice per genotype. A student’s t test was used to calculate p values for WT versus *Mefv* ^−/−^ cultures. #, p<0.05; *, p≤0.01 **, p≤0.001, ***, p<0.0001.

To determine if a loss of pyrin modulates inflammasome-mediated IL-1β production, WT and *Mefv* ^−/−^ rpMΦ were exposed to a variety of elicitors that induce NLRP3 inflammasome-dependent IL-1β production. Cells were pre-treated with LPS to induce IL-1β transcription and then stimulated with one of the following NLRP3 inflammasome elicitors: titanium dioxide (TiO_2_), aluminum hydroxide (Alum), calcium pyrophosphate dehydrate crystals (CPPD), silica, or ATP [6], [33]. IL-1β and IL-6 levels in the cell supernatants were determined by ELISA. We also activated cells with muramyl dipeptide (MDP) and MDP with TiO_2_. MDP alone and MDP plus TiO_2_ were reported to activate both NLRP1 and NLRP3 inflammasomes [7], [33]. However, recent evidence suggests that MDP activates only the NLRP3 inflammasome [29]. As expected, treatment of cells with compounds that trigger assembly of the NLRP3 inflammasome resulted in substantial increase in the levels of extracellular IL-1β, in comparison to cultures treated with LPS alone. In addition, in all cases, higher IL-1β cytokine levels were measured in supernatants collected from *Mefv* ^−/−^ cultures compared to those collected from similarly treated WT cultures. The magnitude of the increase in the levels of IL-1β in *Mefv* ^−/−^ cultures compared to controls varied depending on the stimulus, ranging from an increase of 1.5-fold in cells exposed to MDP and silica to an increase of almost 5-fold in the response of *Mefv* ^−/−^ cells to Alum (Figure 3*A*). No difference was observed in the levels of IL-6 present in the samples from WT and *Mefv* ^−/−^ cultures (Figure 3*B*). Both mature and pro-IL-1β can be released into the supernatant after cell death, and most ELISAs do not bind exclusively to the mature protein. It is therefore possible that the increase in IL-1β in samples collected from *Mefv* ^−/−^ cultures reflects an increase in cell death. To address this question, we measured levels of LDH in the supernatant. Many of the agents used to stimulate NLRP3 assembly decrease cell viability [34], and the magnitude of this decrease was dependent on the agent used to elicit the inflammasome, but viability was not influenced by the absence of pyrin: LDH levels did not differ significantly between *Mefv* ^−/−^ and WT macrophage cultures (Figure 3*C*).

### Negative Regulation of IL-1β Production is not Limited to the NLRP3 Inflammasome

**Figure 4:**
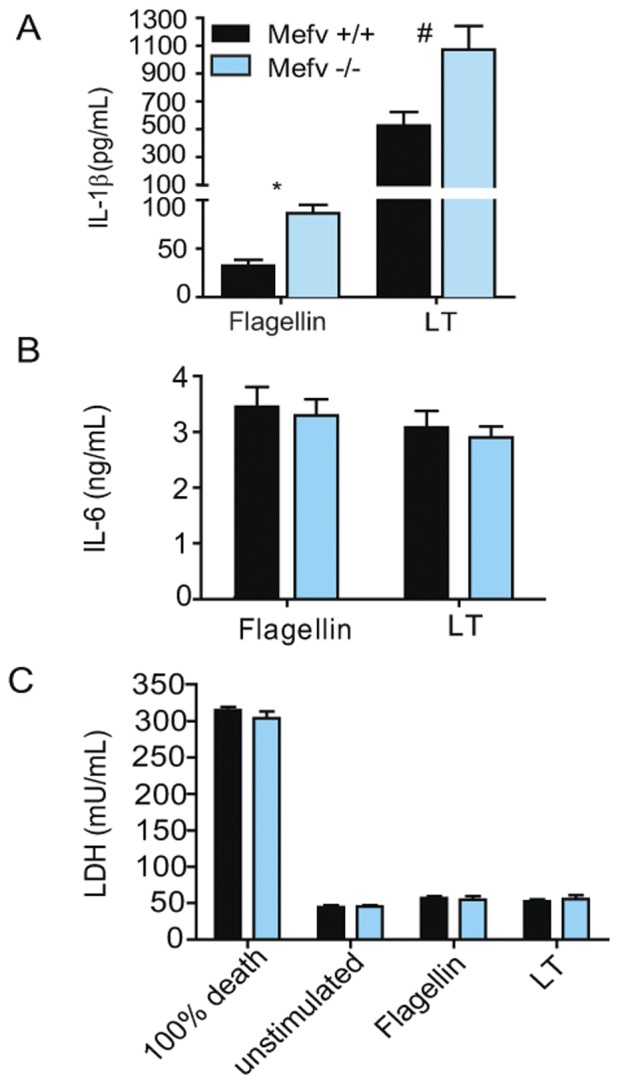
A loss of pyrin causes increased IL-1β release in response to NLRC4 and NLRP1b stimuli. rpMΦs from *Mefv* ^+/+^ and *Mefv* ^−/−^ littermate mice were exposed to LPS and the indicated stimuli as indicated in the method section. The concentration of IL-1β, *A*, and IL-6, *B*, cytokines in cell culture supernatants was determined by ELISA. *C*, LDH protein levels detected in cell supernatants. The total protein of cell lysates was measured to verify equivalent plating of cells. LT, lethal toxin of *Bacillus anthracis*. n = 6 mice per genotype. Results are representative of at least 4 independent experiments. A student’s t test was used to calculate p values for WT versus *Mefv* ^−/−^ cultures. #, p<0.05; *, p≤0.01.

CASP-1 activation and IL-1β release is also observed after exposure of macrophages to agents that mediate the assembly of NLRC4 and NLRP1b -containing inflammasomes [7]. We therefore asked whether a loss of pyrin impacts only NLRP3-mediated IL-1β release or whether it also modulates IL-1β release secondary to assembly of these inflammasomes. WT and *Mefv* ^−/−^ rpMΦ were treated with LPS and exposed to either flagellin, known to elicit the NLRC4 containing inflammasome or *Bacillus anthracis* (anthrax) lethal toxin (LT), activating NLRP1b containing inflammasome in sensitive strains. Significantly higher levels of IL-1β were observed in samples collected from cultures of *Mefv* ^−/−^ macrophages compared to similarly treated WT controls (Figure 4*A*). In contrast, IL-6 and LDH levels in supernatants collected from WT and *Mefv* ^−/−^ cultures were similar (Figure 4*B and C*).

### CASP-1 Activity is not Elevated in *Mefv*-deficient Macrophages

**Figure 5:**
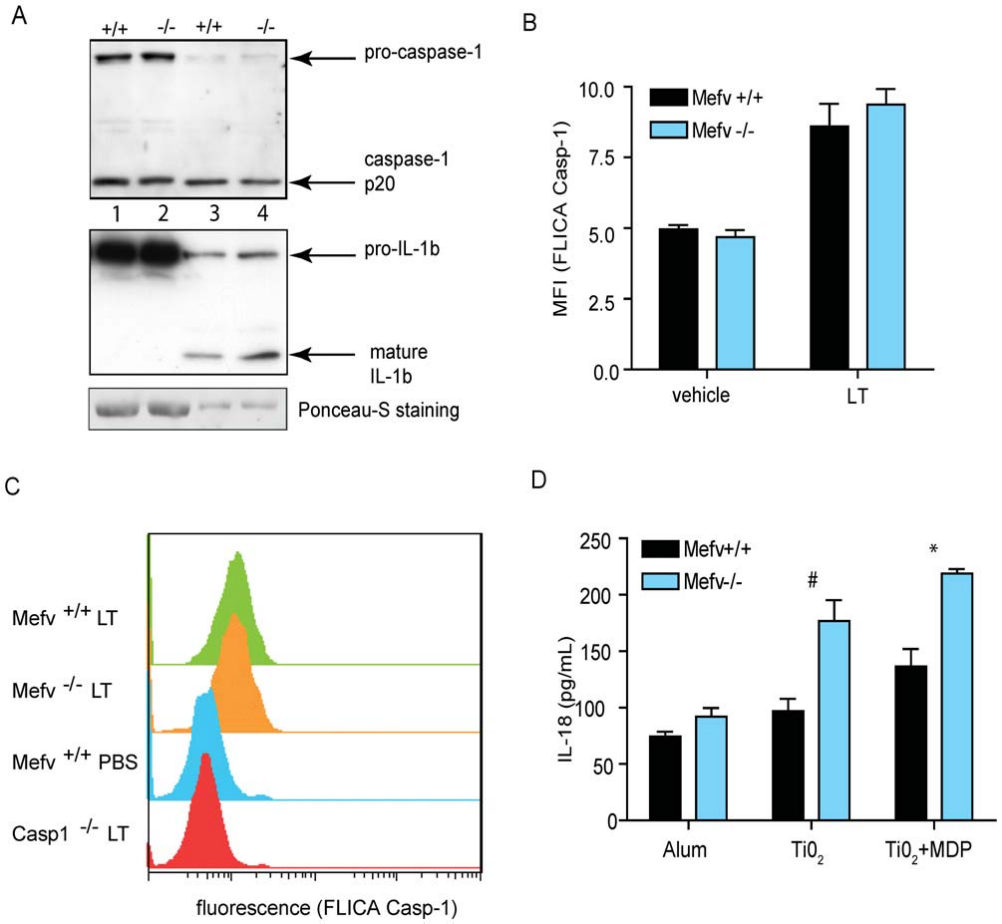
Caspase-1 (CASP-1) activity is not elevated in *Mefv^−/−^* macrophages. *A*, rpMΦs from *Mefv* ^+/+^ and *Mefv* ^−/−^ littermate mice were exposed to LPS and ATP as indicated in the method section. Levels of caspase-1 (upper panel) and IL-1β (middle panel) were detected by western blot in cell lysates (lanes 1 and 2) and cell culture supernatant (lanes 3 and 4). An equivalent amount of total protein was loaded in each lane. The amount of protein in the lysate and supernatant was verified by staining with Ponceau S, major protein band is shown (lower panel). *B* and *C*, *Mefv* ^+/+^, *Mefv* ^−/−^, and *Casp1^−/−^* mice were treated with 10 µg of lethal toxin (LT) or PBS vehicle alone by intratracheal instillation and bronchoalveolar lavage (BAL) was collected at 2 h after treatment. Cells from the BAL were stained with fluorescently labeled CASP-1 inhibitor (FLICA), FAM-YVAD-FMK. Cells were analyzed via flow cytometry. *B*, Data is expressed as the mean of the fluorescence intensity (MFI). *C*, A representative plot of the fluorescence intensity of LT or vehicle-treated cell populations. *D*, rpMΦs from *Mefv* ^+/+^ and *Mefv* ^−/−^ littermate mice were treated with LPS and the indicated stimuli as in Figures 3 and 4. The concentration of IL-18 in cell culture supernatants was determined by ELISA. A student’s t test was used to calculate p values for *Mefv* ^+/+^ versus *Mefv* ^−/−^ cultures. #, p<0.05; *, p<0.01.

Thus far, our results are consistent with two hypothetical models. In the first, pyrin affects pro-IL-1β processing by inhibiting activity of the inflammasome complexes. This could occur either by regulating an upstream event common to NLRP3, NLRP1b, and NLRC4 inflammasome assembly or by directly inhibiting assembly and activity of the inflammasome complexes themselves. In the second model, pyrin regulates release of mature IL-1β from macrophages subsequent to inflammasome activity. To address these possibilities, we asked if inflammasome-stimulated CASP-1 activity was altered in *Mefv^−/−^* macrophages. Macrophages were treated with LPS/ATP, and levels of pro-caspase-1 and processed CASP-1 (p20) were assessed by western blot analysis. As expected, LPS/ATP induced processing of CASP-1, which could be detected in cell lysates and in media collected from the macrophage cell cultures. The levels of processed CASP-1, and the levels of pro-caspase-1 in the cell lysates, did not differ between wild type and *Mefv* ^−/−^ cultures. CASP-1 activation was accompanied by processing and release of mature IL-1β, as detected by western blot in the culture media of the macrophages. Similar to measurements of IL-1β shown in Figure 3A, an increased amount of IL-1β was detected in the media from *Mefv^−/−^* cells compared to wild-type cells (Figure 5*A*). To further address CASP-1 activation, the activity of CASP-1 was determined by using a fluorescently labeled inhibitor for CASP-1 (FAM-YVAD-FMK). Mice were exposed to *Bacillus anthracis* lethal toxin (LT) or PBS vehicle by intratracheal (*i.t.*) instillation. Bronchoalveolar lavage fluid (BAL) was collected at 2 h post-treatment, and cells of the BAL were assessed by FACS analysis to detect CASP-1 activity. CASP-1 activity increased following LT treatment in both wild-type and *Mefv^−/−^* alveolar macrophages, and furthermore, CASP-1 activity did not differ between wild-type and *Mefv^−/−^* mice. To ensure the specificity of our assay for measuring CASP-1 activity, *Casp1^−/−^* mice were also evaluated, and as expected, no CASP-1 activity was induced in these mice (Figure 5*C*). Next, we addressed whether or not the inhibitory effect of pyrin extended to IL-18, another IL-1 family cytokine that is processed and released from cells through pathways common to IL-1β. *Mefv^−/−^* macrophage cultures exposed to inflammasome stimuli had elevated levels of IL-18 protein in the cell culture supernatant compared to wild-type macrophages, thus mimicking IL-1β levels (Figure 5D).

## Discussion

We describe here the generation of a mouse in which the entire pyrin gene is excised. Although a more difficult mutation to generate, this strategy ensures the loss of not only the primary *Mefv* transcript but also transcripts initiated from internal promoters and splice variants from the *Mefv* locus. Thus, proteins containing all or a subset of the many functional domains of pyrin cannot be generated from this modified locus. Using these mice, we show a marked change in the response of primary macrophages to a number of inflammatory stimuli; specifically, a significant increase in the release of IL-1β observed in cultures derived from *Mefv* ^−/−^ mice in comparison to WT mice.

Since the discovery of the genetic lesions which cause FMF, numerous models have emerged that attempt to incorporate information gleaned from the genetic and clinical characteristics of the disease, from the structure of the protein and its pattern of expression, and from studies of the gene in model systems. One model suggests that pyrin is a substrate for CASP-1, and the N-terminal fragment of pyrin potentiates inflammation by interacting with proteins of the NF-κB pathway. An increased ratio of cleaved to intact protein was detected in clinical samples from FMF patients [30]. However, other investigators have not observed increased NF-κB-dependent luciferase activity following overexpression of full-length *MEFV* [16], [20]. Our studies of mouse macrophages lacking pyrin do not support a role for pyrin in activity of the NF-κB pathway, although more extensive studies are required. An increase in expression of NF-κB sensitive transcripts was not observed in *Mefv* ^−/−^ cells in a naïve state or after exposure to LPS. As expected, *i.p.* injection of LPS resulted in an acute peritonitis characterized by a rapid drop in body temperature and accumulation of inflammatory cells in the peritoneal cavity. However, again, no difference was observed in the response of the mutant mice compared to control littermates.

The phenotype of the *Mefv* null mice differs in some aspects from those reported previously for mice carrying a disrupted *Mefv* locus. Macrophages from these mice were shown to release more IL-1β than wild type cells. In addition, these animals displayed an exaggerated cryogenic response to low-dose LPS, and an increase in morbidity upon exposure to high-dose LPS or LPS and D-Gal. An increase in the number of inflammatory cells recovered from peritoneal lavage fluid was observed in these mice after exposure to thioglycolate. Furthermore, apoptosis was impaired in macrophages collected from animals homozygous for the targeted locus [14]. In contrast, we did not observe an increase in the release of IL-1β from *Mefv* ^−/−^ cells treated with LPS alone, and the response of the *Mefv* ^−/−^ animals to LPS delivered *i.p.* could not be distinguished from that of the controls. We also failed to detect any change in the survival of *Mefv* null cells: no difference in the rate of cell death was observed in cultured macrophages, or in the number of cells surviving after stimulation of these cultures with inflammatory mediators. In addition, *ex vivo* survival of recruited peritoneal neutrophils lacking pyrin was not altered (data not shown). One explanation for the differences between the mutant *Mefv* line characterized in this earlier report and the line described here is that in the former, a *Mefv* transcript and a truncated pyrin protein were created by the targeted allele. In fact, the level of this truncated protein, based on western analysis, appeared to be higher than that observed for the native protein. It is possible that this truncated protein conferred unique functions not observed in mice carrying a null allele.

While we did not observe an increase in the IL-1β release by macrophages in response to LPS alone by cells lacking pyrin, increased levels of IL-1β were detected in the culture media after stimulation of these mutant cells with agents that lead to inflammasome assembly. The increase in IL-1β levels was observed in supernatants collected from cells stimulated with agents known to trigger assembly of NLRP3, NLRP1b, and NLRC4 inflammasomes. Increased release of IL-1β from cells in response to all of the tested inflammatory stimuli is inconsistent with a model in which pyrin modulates IL-1β production by regulating the assembly of a specific inflammasome, such as the NLRP3 inflammasome. It is unlikely that pyrin acts to block ASC or CASP-1 association with the inflammasome, because no alteration in CASP-1 activity was observed between *Mefv* ^−/−^ and wild-type cells (Figure 5*A–C*). Our results differ from studies carried out with a human monocyte/macrophage cell line and PBMCs in which silencing of the *MEFV* transcript suppressed secretion of IL-1β [25], [26], and from transfection studies in which overexpression of pyrin increased CASP-1 activity and IL-1β release [20]. However, our results are consistent with models in which pyrin acts as a negative regulator of innate immune pathways, and with a genetic model in which pyrin mutations causing FMF represent loss-of-function mutations. In opposition to the studies mentioned above, silencing of *MEFV* has been shown to increase IL-1β release from a human monocyte/macrophage cell line [15], [17]. This model is also supported by transfection studies in which wild-type pyrin inhibited pro-IL-1β processing in macrophage cell lines, whereas the FMF-associated alleles proved less active in these assays [14], [15].

It is becoming increasingly apparent that production of IL-1β is regulated at many different levels, and that the relative importance of these mechanism(s) is likely dependent on many factors including the cell type, its local environment, and the nature of the stimuli [35]. For example, recently, induction of autophagy by rapamycin was reported to result in degradation of pro-IL-1β and reduced secretion of IL-1β [36]. Interestingly, in one study, examination of PMNs from FMF patients revealed a gene expression pattern consistent with impaired basal autophagy [37], raising the possibility that increased IL-1β levels in FMF patients and the increase in IL-1β release by the *Mefv* ^−/−^ macrophages reflect a defect in regulation of cellular levels of pro-IL-1β. Many other means by which pyrin could modulate release of IL-1β are apparent. For example, alteration in the uptake of particulate matter and/or decrease in lysosomal stability could increase IL-1β maturation in response to many stimuli [7]. In models of gout, crystal-induced IL-1β production has been shown to be affected by Cathepsin B inhibitors that prevent lysosomal instability [38], and crystal-induced IL-1β production can be blocked by colchicine, a prophylactic drug for FMF patients [39]. Alternatively, pyrin may regulate the trafficking or release of vesicles containing mature IL-1β and IL-18, a process that is poorly understood [5], [40], [41]. This hypothetical model is consistent with our finding that levels of released IL-1β and IL-18 protein are higher in *Mefv^−/−^* cultures compared to wild-type despite similar levels of CASP-1 activity in *Mefv^−/−^* and control macrophages. In previous studies, pyrin was shown to interact both directly and indirectly with cytoskeletal elements, which are necessary for vesicle trafficking and release [42], [43]. Pyrin has been shown to bind directly to microtubules [44] and to actin [44], [45]. Furthermore, it was previously shown that VASP and Arp3 proteins, which are important in actin polymerization, co-immunoprecipitate with pyrin [45]. Pyrin also binds to proline-serine-threonine phosphatase interacting protein 1 (PSTPIP1) [27], [46], and PSTPIP1 binds to PTP-PEST, which is a scaffolding protein that associates with actin through the protein WASP [42]. Mutations in PSTPIP1 are associated with PAPA syndrome, an autoinflammatory disorder similar to FMF, further supporting a functional role of this pathway in inflammation. Together, these data support a hypothesis that pyrin regulates trafficking or release of vesicles containing IL-1β and IL-18. Further studies will be required to identify at which of these many steps pyrin acts to limit release of IL-1β and IL-18.

During the preparation of this manuscript, an additional mouse line carrying a mutant *Mefv* ^−/−^ gene has been reported. No difference in the release of IL-1β by macrophages after treatment with LPS or LPS and ATP, after infection with *Salmonella typhimurium*, or after transfection of DNA was reported [24]. A number of explanations for these differences are possible. First, while our studies use mature peritoneal macrophages which express high levels of pyrin (Figure 1*A*), Chae, et al. examine IL-1β production in bone marrow-derived macrophages. As cultured bone marrow-derived macrophages express very low levels of pyrin, the impact of the loss of this protein on IL-1β processing and/or release might not be as apparent with this cell type as on comparison of cells with higher levels of expression. In addition, the two studies use mice of differing genetic backgrounds: those reported here carried out entirely with co-isogenic 129S6 mouse lines, while those of Chae and colleagues used N6 B6.129 animals. It is possible that the regulatory role of pyrin might be more apparent on some genetic backgrounds. In particular, caspase-4 is not functional in 129 genetic background.

The demonstration here that loss of pyrin can result in increase in IL-1β release is consistent with the original designation of FMF as a recessive genetic disorder. However, perhaps for no other autoinflammatory disorder is the validity of extrapolating results from mouse studies more subject to scrutiny than for FMF. Pyrin encoded by the mouse gene lacks the C-terminal domain, termed B30.2, that is present in the human protein. This complicates comparison of gene function, not only because numerous functions including interaction with CASP-1, NLRPs1-3, and IL-1β have been assigned to this domain [17], but also because the majority of the FMF mutations are located in this C-terminal region [14]. In addition, major differences in the regulation of the gene have been noted between human and mouse: while the mouse gene is induced by IL-4 [14], this cytokine inhibits expression of the human ortholog [47]. These differences raise the possibility that one or more functions of pyrin is unique to the human protein and is therefore difficult to model in the mouse. Thus, until novel methods and reagents are developed for addressing this limitation, we can only ascertain that pyrin can act to limit the activity of proinflammatory pathways, namely the release of IL-1β by macrophages, in at least some species.
